# CpG island-mediated global gene regulatory modes in mouse embryonic stem cells

**DOI:** 10.1038/ncomms6490

**Published:** 2014-11-18

**Authors:** Samuel Beck, Bum-Kyu Lee, Catherine Rhee, Jawon Song, Andrew J. Woo, Jonghwan Kim

**Affiliations:** 1 Department of Molecular Biosciences, The University of Texas at Austin, Austin, Texas 78712, USA; 2 Texas Advanced Computing Center, The University of Texas at Austin, Austin, Texas 78758, USA; 3 School of Medicine and Pharmacology, Royal Perth Hospital Unit, The University of Western Australia, Perth, WA 6000, Australia; 4 Institute for Cellular and Molecular Biology, The University of Texas at Austin, Austin, Texas 78712, USA; 5 Center for Systems and Synthetic Biology, The University of Texas at Austin, Austin, Texas 78712, USA

## Abstract

Both transcriptional and epigenetic regulations are fundamental for the control of eukaryotic gene expression. Here we perform a compendium analysis of >200 large sequencing data sets to elucidate the regulatory logic of global gene expression programs in mouse embryonic stem (ES) cells. We define four major classes of DNA-binding proteins (Core, PRC, MYC and CTCF) based on their target co-occupancy, and discover reciprocal regulation between the MYC and PRC classes for the activity of nearly all genes under the control of the CpG island (CGI)-containing promoters. This CGI-dependent regulatory mode explains the functional segregation between CGI-containing and CGI-less genes during early development. By defining active enhancers based on the co-occupancy of the Core class, we further demonstrate their additive roles in CGI-containing gene expression and cell type-specific roles in CGI-less gene expression. Altogether, our analyses provide novel insights into previously unknown CGI-dependent global gene regulatory modes.

## Abstract (web summary)

CpG islands are high GC content DNA elements that surround the majority of transcriptional start sites in eukaryotes. Here, the authors analyse over 200 genomic data sets to provide new insight into global CpG islands-dependent regulatory mechanisms in differentiated and pluripotent stem cells.

## Graphical abstract

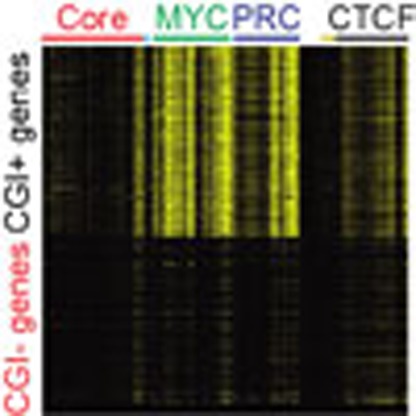

The precise control of global gene expression programs is governed by multi-layered regulatory steps that include transcriptional^1^,^2^ and epigenetic^3^,^4^ regulation. Both processes are mediated by the combinatorial actions of hundreds of trans-acting DNA-binding proteins (DBPs)^5^ and numerous target cis-regulatory elements^1^. Recent advances in high-throughput technologies, such as massive parallel sequencing in combination with chromatin immunoprecipitation (ChIP-seq) and gene expression profiling (RNA-seq), have enabled researchers to identify unbiased genome-wide interactions between DBPs and their genomic target loci, and further enlighten the functional outcomes. Not only the acquisition but also comprehensive analyses of large data sets have become crucial for elucidating the elaborate regulatory mechanisms of global gene expression^6^.

Transcriptional regulation has been suggested as one of the key steps in maintaining the identity of embryonic stem (ES) cells represented by two terms: self-renewal and pluripotency^7^. In addition to the identification of core transcription factors (TFs), such as OCT4, SOX2 and NANOG^8^,^9^,^10^,^11^, subsequent studies on the expansion of transcriptional regulatory circuitry have suggested that global gene expression regulation in ES cells is achieved by functionally separable regulatory sub-modules^12^,^13^. This modular regulation requires close interactions between multiple DBPs including TFs and chromatin regulators and a distinct set of their chromosomal targets. This cooperative modular action not only underscores the importance of studying multiple DBPs within specific regulatory units but also elucidates the significance of the systematic identification of their genomic targets and associated chromatin or DNA modification states.

CpG islands (CGIs) are DNA elements with high GC contents, existing mostly without DNA methylation, and surrounding >60% of the transcriptional start sites (TSSs) of eukaryotic genes^14^,^15^. Early genomic sequencing analysis has shown that CGIs exist invariably on almost all housekeeping genes and less frequently on tissue-specific genes^16^,^17^,^18^. Although CGIs have been previously suggested as one of the important regulatory elements influencing the transcriptional activity of many genes^19^,^20^, only a few CGI-specific binding DBPs, such as KDM2A, CFP1 and TET1 (refs 21, 22, 23), have recently been identified. Therefore, the functional significance of CGI-mediated transcriptional and epigenetic regulations is only beginning to be understood. Comprehensive and systematic approaches to understanding the differences between CGI-containing (CGI+) and CGI-less (CGI−) promoters in their associated gene expression patterns, global regulatory mechanisms and functional implications have not been clearly demonstrated.

In order to elucidate how DBPs interact with and precisely control the cis-regulatory elements over gene expression programs in ES cells especially within the context of CGIs, we conducted an integrative analysis of publicly available data sets with our new data incorporating the DBP occupancies, histone modification signatures, chromatin accessibility and DNA methylation. The tested DBPs were classified into multiple sub-groups based on their target co-occupancy. Strikingly, we found predominant regulation in CGI+ promoters by the MYC and polycomb repressive complex (PRC)-related DBPs. This CGI-dependent regulatory mode further explains the functional segregation between the CGI-containing and CGI-less genes during early embryonic development. We also defined the active enhancers by the Core class DBPs, and showed that these enhancers regulate cell type-specific gene expression programs in ES cells. In summary, our findings provide novel insights into CGI-dependent global gene regulatory modes in ES cells, characterized by the general regulation of CGI+ promoters by the MYC and PRC classes, and the tissue-specific regulation of CGI− genes by enhancer binding Core class DBPs.

## Results

### Co-occupancy guided classification of DBPs

**Figure 1:**
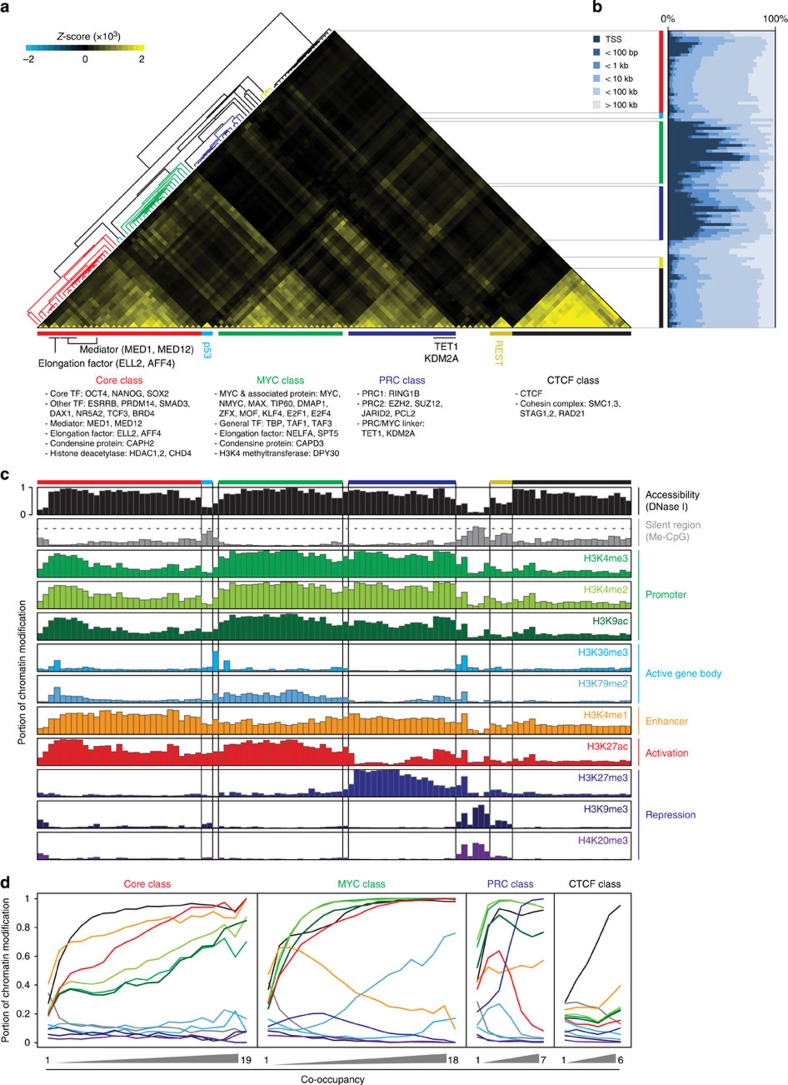
Co-occupancy guided identification of DBP classes and their associated chromatin status. (**a**) Co-occupancy guided classifications of DBPs. Heatmap shows six distinctive clusters (colour-coded bars) from an unsupervised hierarchical clustering of co-occupancies for 105 DBPs. Representative DBPs and their putative functions are shown below. The extent of co-occupancy between two DBPs was monitored with the deviation of actual co-occupancy from randomized expectation (*Z*-score, see Methods section). Red: Core class (19 DBPs, 29 experiments), blue: PRC class (7 DBPs, 21 experiments), green: MYC class (18 DBPs, 22 experiments), black: CTCF class (6 DBPs, 21 experiments), cyan: P53 class (single DBP, 2 experiments) and yellow: REST class (single DBP, 4 experiments). (**b**) Distance distribution of DBP-binding sites from transcriptional start sites (TSSs). DBPs are listed in the same order as in Fig. 1a. Note that DBP-binding sites in the PRC and MYC classes are generally enriched nearby TSSs, while the binding sites of the Core and CTCF classes are further away from TSSs. (**c**) Association of each DBP class with a unique chromatin status. Each colour-coded bar graph, excluding me-CpG, shows the portion containing a specific histone mark or DNase I hypersensitivity (*y* axis) within the given DBP-binding sites (*x* axis, same order as in Fig. 1a). Me-CpG (grey) indicates the average portion (*y* axis) of CpG methylation within the given DBP-binding sites (*x* axis). Genome-wide average portion of CpG methylation (0.6474) is shown with a grey dashed line. (**d**) Association of increased co-occupancy of DBPs within the class with specific histone marks. The portions containing chromatin marks or percentage of CpG methylation (*y* axis) within the centre (300 bp) of the region co-occupied by indicated number of DBPs (*x* axis) are plotted with different colour as indicated in Fig. 1c.

Previous studies on ES cells have shown the cooperative regulation of DBPs on their common targets^7^,^13^,^24^. In order to obtain further insights into DBP-mediated transcriptional regulation in ES cells, 166 genome-wide DBP occupancy data sets were initially tested (157 publicly available ChIP-seq and 9 newly acquired from the bioChIP-seq^24^; Supplementary Dataset 1), and we selected 105 high-quality data sets to classify DBPs based on their target co-occupancy on the genome (Supplementary Dataset 2 and Methods section). As a result, we observed six distinct DBP classes and named them based on the representative factors within each class (Core, PRC, MYC, CTCF, REST and P53; Fig. 1a). The Core, PRC and MYC classes were similar to our prior observations^12^ with additional DBPs, and we identified three new classes (CTCF, REST and P53; Fig. 1a and Supplementary Dataset 2). Notably, the MYC class included the elongation factors^25^ and general TFs, in addition to other previously known DBPs^12^,^13^. The CTCF class consisted of insulator protein, CTCF and cohesion complex members. In addition to four major classes (Core, PRC, MYC and CTCF; Fig. 1a), two small classes, P53 and REST, showed unique binding patterns, indicative of their distinct roles in ES cells (Fig. 1a). We noticed that some DBPs shared targets across multiple classes, for instance, TET1 (ref. 22) and KDM2A (ref. 21) shared targets with DBPs in the PRC and MYC classes. The mediators^26^,^27^ and elongation factors^28^ in the Core class also shared binding sites with the members of the MYC class (Fig. 1a and Supplementary Fig. 1a), implying that these are functionally important in connecting multiple classes of DBPs.

As shown in Supplementary Fig. 1b,c, we observed a positive correlation between the co-occupancy and the strength of the DBP binding only within the same class of DBPs, suggesting that DBPs from the same class function cooperatively. As previously reported, the PRC and MYC classes generally occupy sites near TSSs, whereas the Core and CTCF classes generally localize further away from the TSSs, suggesting a unique proximal or distal regulatory mode for each class (Fig. 1b). Since the target occupancy of DBPs is often associated with specific chromatin marks^29^,^30^,^31^, we examined the association between the co-occupancy of DBPs and 10 histone modification signatures, chromatin accessibility (DNase I hypersensitivity regions, DHRs) or DNA methylation (Supplementary Dataset 3 and Fig. 1c,d). The Core class targets were highly enriched in DHRs with a putative enhancer histone signature, H3K4me1 (ref. 31). The MYC class preferentially bound to DHRs with the active histone markers, H3K4me3 and H3K27ac. The binding regions of the PRC class harboured both active (H3K4me3) and repressive (H3K27me3) markers as previously reported^19^,^29^. Interestingly, we found that the PRC class binding regions are mainly within DHRs, implying that unlike in previous reports^32^,^33^, chromatin condensation is not an absolute requirement for PRC-mediated gene repression. The binding sites of all four major classes are enriched within the DHRs with the depletion of DNA methylation (Fig. 1c,d). Taken together, our results show that DBPs within the same class share many common regulatory features and that each class may have a distinct role in global gene regulation.

### The MYC and PRC classes predominantly occupy CGI+ promoters

**Figure 2:**
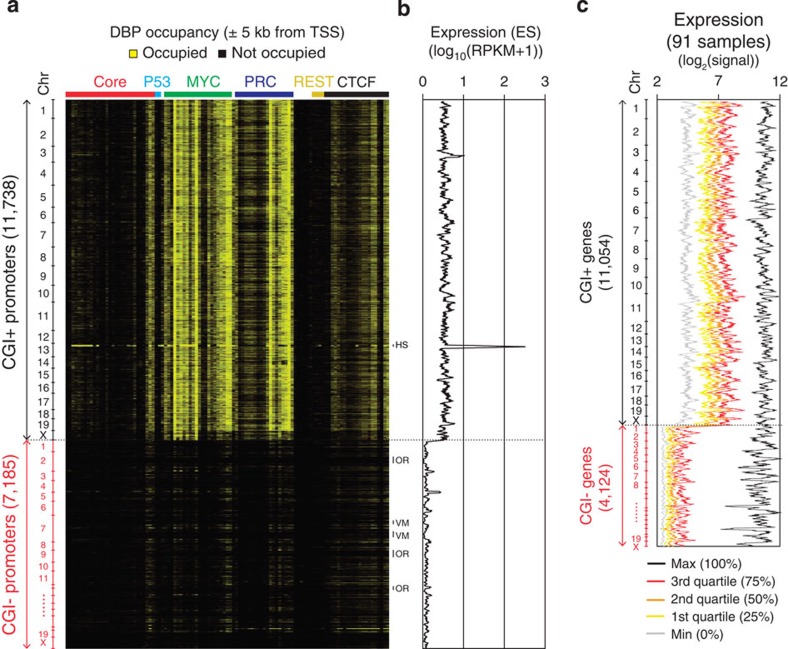
Predominant regulation of the MYC and PRC classes on CGI+ promoters. (**a**) Heatmap presenting DBP occupancies (same order as in Fig. 1a) within±5 kb from TSSs of CGI+ and CGI− genes (vertical, chromosomal order). Gene clusters showing distinct binding patterns are shown on the right as follows. HS, histone gene cluster; OR, olfactory receptor gene cluster; VM, vimentin gene cluster. (**b**) The expression value of each gene in ES cells is plotted using a moving average (window size: 64, bin size: 1; genes are in chromosomal order as in Fig. 1a) across CGI+ and CGI− genes. Expression levels are shown in log_10_(RPKM+1) scale. RPKM, reads per kilobase per million. (**c**) Moving average plots (window size: 64, bin size: 1; genes are in chromosomal order) showing global gene expressions of 91 different tissues or cell lines (http://biogps.gnf.org)^34^. Expression values are ranked for each gene, and maximum, quartiles and minimum values among samples are plotted with different colours as indicated.

As shown in Fig. 1a, our analysis revealed that previously reported CGI-binding proteins, such as KDM2A (ref. 21) and TET1 (ref. 22), share targets with the MYC and PRC classes. In addition to a prior report that suggested the CGI-dependent regulation of PRC1/2 (ref. 29), our data imply that CGIs may be equipped to control local gene activity in combination with specific DBPs^15^. Since the MYC and PRC classes tend to occupy proximal promoter regions among the multiple DBP classes we defined (Fig. 1a,b), we hypothesized that the major roles of the MYC and PRC classes are restricted to CGI+ promoters. To test this hypothesis, we examined the extent of the global target occupancy of DBPs in all classes on the CGI+ and CGI− promoters (Fig. 2a). Markedly, most DBPs in the MYC and PRC classes showed a strong occupancy on the CGI+ promoters only (Fig. 2a and Supplementary Fig. 2). Subsequent tests for global gene expression showed that the gene activity under the control of the CGI+ promoters (hereafter, CGI+ genes) is significantly higher than the activity of genes with CGI− promoters (CGI− genes; Fig. 2b). Further testing of the gene expression profiles from 91 tissue samples^34^ revealed that the overall levels of the CGI+ genes are much higher than the levels of the CGI− genes (Fig. 2c). These results suggest a general ‘on’ state of the CGI+ genes in ES cells. The expression of the CGI− genes, however, was skewed to a minimum value in most samples, while being selectively active in a few samples, indicating a general ‘silent’ and a context-dependent ‘on’ state of the CGI− genes. Taken together, these findings strongly suggest two CGI-dependent modes of global gene regulation: one for CGI+ genes and the other for CGI− genes.

### The MYC and PRC classes determine the activity of CGI+ genes

**Figure 3:**
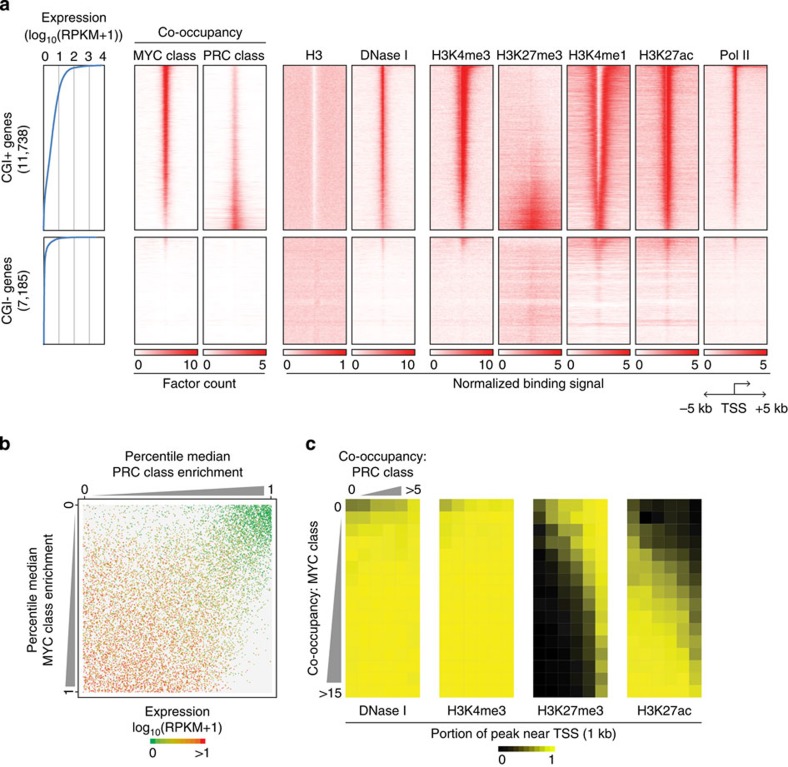
The MYC and PRC classes reciprocally determine the activity of CGI+ genes. (**a**) Profiles of the MYC and PRC class co-occupancies, histone marks, DNase I hypersensitivity signal and Pol II occupancy within±5 kb from the TSSs of CGI+ and CGI− genes. All protein-coding genes are sorted by their expression values (left panel, blue lines) and ChIP-seq profiles are shown as heatmaps. (**b**) A dot plot showing the correlation between the combinatorial occupancy of PRC (*x* axis) and MYC (*y* axis) class DBPs and corresponding CGI+ gene expression. Each dot indicates an individual gene. Total 11,738 genes were shown (see Methods section for detail). (**c**) Heatmaps showing the MYC and PRC class co-occupancy and their associated chromatin status near the TSSs of CGI+ genes. Each cell in the heatmaps indicates the average portion of the indicated chromatin mark within 1 kb regions of the TSSs (±500 bp from TSS) co-occupied by the indicated number of DBPs from the MYC (vertical) and PRC (horizontal) classes.

We found that up to 99.4% (11,667 out of 11,738) of all CGI+ genes are occupied by at least one factor from either the MYC or PRC class, while <10% of the CGI− genes are their targets (Fig. 3a and Supplementary Fig. 3a). As expected, the activity of the CGI+ genes showed a positive correlation with MYC class co-occupancy and a negative correlation with the PRC class (Fig. 3a,b). In accordance with this observation, the active CGI+ genes harboured high levels of MYC-associated active histone markers, such as H3K4me3 and H3K27ac, whereas the inactive CGI+ promoters harboured repressive H3K27me3 markers (Fig. 3a,c, see also Fig. 1c,d and Supplementary Fig. 4a). Only a small number of highly active CGI− genes showed a marginal level of MYC class binding with active histone markers (Fig. 3a and Supplementary Fig. 4a). Collectively, these results suggest that there is selective regulation of CGI+ genes by the MYC and PRC classes. In agreement with prior reports of non-linear target gene amplification^35^,^36^ and RNA polymerase II (Pol II) release of Myc^25^ as well as the direct inhibition of target gene expression by disassembling the Pol II pre-initiation complex by PRC^37^, our analyses indicate that the activity of nearly all CGI+ genes can be generally explained by the reciprocal regulation between the MYC and PRC classes (Fig. 3b).

### Functional separation between the CGI+ and CGI− genes

**Figure 4:**
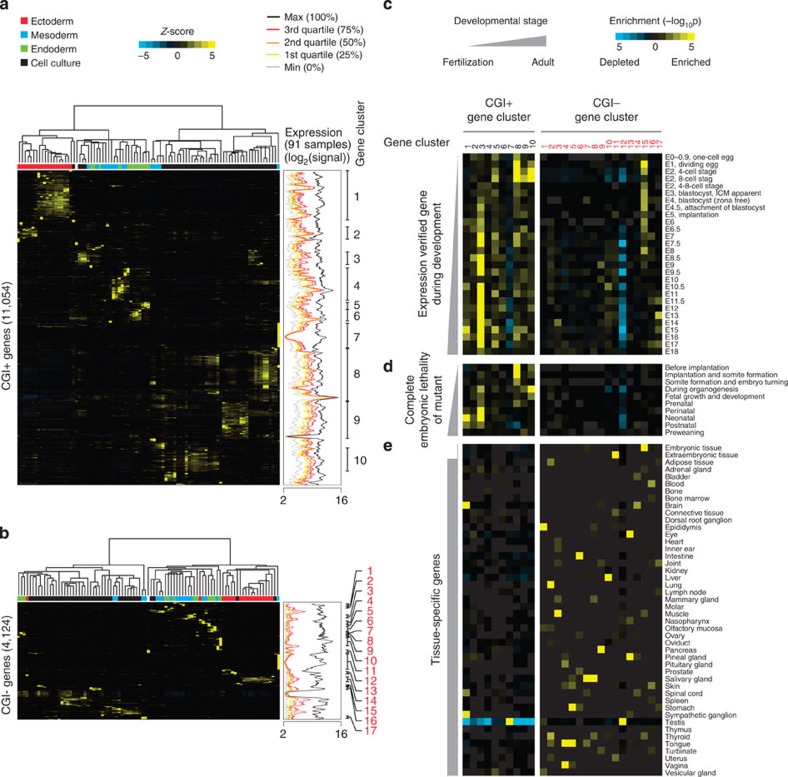
Functional segregation between CGI+ and CGI− genes during early embryo development. (**a**,**b**) Unsupervised hierarchical clustering of gene expression variances (*Z*-scores) for CGI+ (**a**) and CGI− genes (**b**) from 91 tissues or cell lines (http://biogps.gnf.org^34^; left clustering result). For each sample, the putative origin of embryonic germ layer is indicated with a different colour (upper part of heatmap). Moving average plots in the middle (window size: 64, bin size: 1; genes are ordered as in gene cluster results) show global gene expressions of 91 different tissues or cell lines as shown in Fig. 2c. Gene clusters (gene sets) showing a similar pattern of expression variances are defined, and indicated as numbers in black and red (CGI+ and CGI− genes, respectively; right end). (**c**–**e**) Heatmaps showing hypergeometric *P* values calculated by gene set overlap analyses between gene sets from hierarchical clustering (Fig. 4a,b) and expression verified gene sets during embryo development (**c**), gene sets showing complete embryonic lethality upon deletion (**d**) or tissue-specific genes (**e**) (see Methods section for detail).

Our analyses revealed that there are marked differences in global gene activity, DBP occupancy and histone signatures between CGI+ and CGI− genes (Figs 2 and 3). Although more tissue-specific activity of CGI− genes was previously suggested^17^,^38^, the functional segregation between CGI+ and CGI− genes has not been systematically addressed. To address this issue, we looked into gene expression variations across different tissue samples^34^ by performing two independent hierarchical clustering analyses of the CGI+ and CGI− genes (Fig. 4a,b; see Methods section). Among the CGI+ genes, large groups of gene clusters (gene sets; numbers in black) were co-regulated and synchronously activated in the tissues from the same developmental origins (Fig. 4a). In contrast, the clustering of the CGI− genes generated smaller gene clusters (numbers in red) within a limited number of tissues (Fig. 4b).

We then performed gene set overlap tests (see Methods section) to understand the functional implications of the CGI context in developmental stages and tissue specificity, by comparing the gene sets defined from the clustering analyses (Fig. 4a,b) with previously identified active gene sets during development (Gene Expression Database)^39^. The comparison revealed that the gene sets acquired from the CGI+ genes are frequently activated throughout early embryonic development, whereas the gene sets from the CGI− genes mostly remained silent (Fig. 4c). Likewise, the comparison of the gene clusters with the previously known mutant phenotypes (Mouse Genome Informatics, MGI)^40^ showed that the loss of CGI+ genes often led to embryonic lethality (Fig. 4d). However, the comparison with the tissue-specific gene lists in the adult mouse (Unigene) showed that tissue-specific expression is more distinctive in CGI− genes (Fig. 4e). Collectively, our integrative analysis provides clear evidence showing that there are functionally separated CGI-dependent regulatory modes (Figs 2 and 3). Many active CGI+ genes are required for general cellular processes with a basal level of gene activity, whereas the mostly silent CGI− genes are selectively activated in more specialized cell types or tissues.

### The Core class selectively defines active enhancers

Among various strategies for identifying enhancers, co-occupancy mapping of a cluster of TFs has been suggested as the most reliable method with fewer false positives and negatives^41^. Our own analyses also showed that DBPs in the Core class occupy distal regulatory regions with a high level of H3K4me1, the marker used for the chromatin signature of enhancers (Fig. 1c,d). Since the Core class DBPs share many common targets with the factors known to occupy enhancer elements including mediators (MED1 and MED12) as well as co-activators (P300, LSD1 and CHD7) (Fig. 1a and Supplementary Fig. 1a)^27^,^42^, we sought to define the enhancers using the co-occupancy of the Core class DBPs in ES cells.

**Figure 5:**
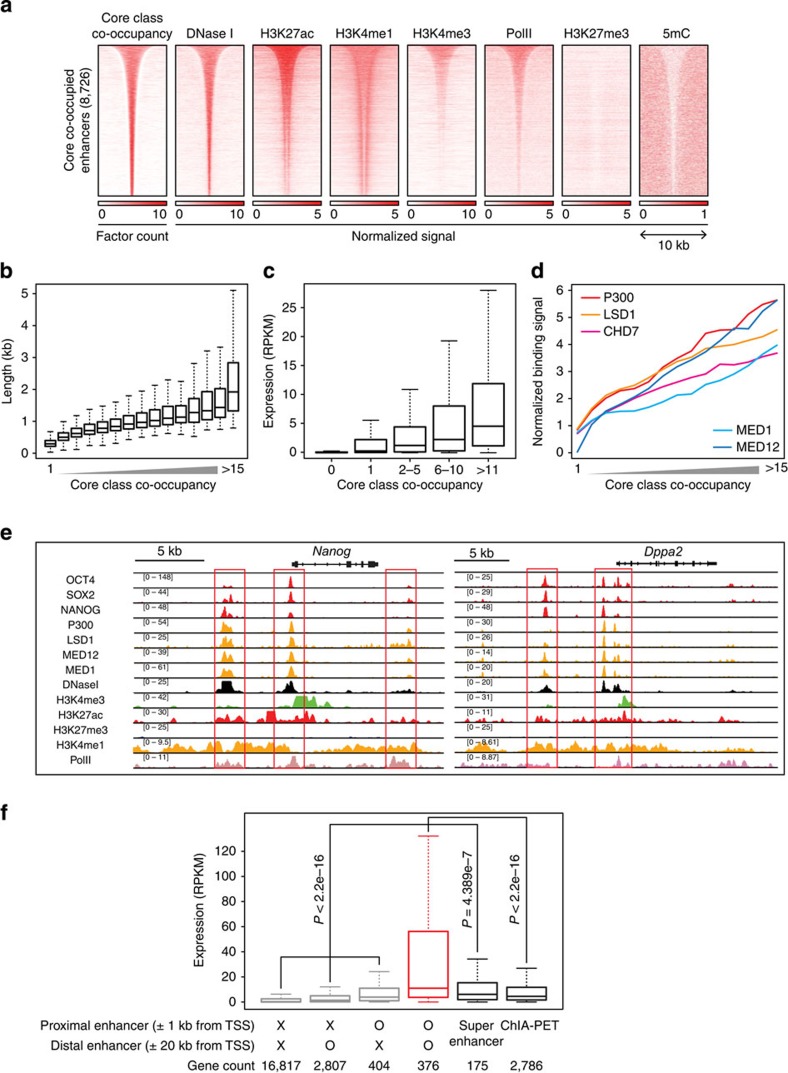
The Core class DBPs define active enhancers in ES cells. (**a**) Profiles showing the co-occupancy of the Core class DBPs, chromatin accessibility (DNase I), Pol II occupancy, histone modification marks and DNA methylation within±5 kb from the centre of the Core class co-occupied (>5 DBPs; Supplementary Dataset 4) enhancers. The enhancers are sorted by the lengths of their co-occupied regions. (**b**–**d**) The correlation between the co-occupancy of the Core class (*x* axis) and the length distribution of the regions co-occupied by the Core class DBPs (**b**), target gene activity (±5 kb) (**c**), and normalized binding signals of co-activators (P300, LSD1 and CHD7) and mediators (MED1 and MED12) (**d**). (**e**) Examples of genes controlled by multiple enhancers. ChIP-seq signal tracks for the Core class DBPs (OCT4, SOX2 and NANOG), co-activators (P300 and LSD1), mediators (MED1 and MED12), Pol II, open chromatin (DNase I) and diverse histone marks are shown. Enhancer elements are highlighted with red rectangular boxes. (**f**) Box plot showing the expression of genes regulated by different types of enhancers; proximal (±1 kb from TSS), distal (±20 kb from TSS) and multiple enhancers (both proximal and distal). ‘O’ and ‘X’ indicate the presence and absence of enhancers in surrounding regions of TSSs, respectively. Expressions of genes regulated by super enhancers^26^ and genes physically interacting with enhancers identified by chromatin interaction analysis with paired-end tagging (ChIA-PET)^48^ are also shown. *P* values were calculated from Wilcoxon signed-rank test between two groups.

We mapped the enhancers along with a number of the co-occupied Core class TFs. To determine the appropriate threshold for mapping reliable enhancers, we compared P300 signals, a representative enhancer mark, from the enhancers we defined based on the co-occupancy of the Core class DBPs and the enhancers defined by other enhancer annotation methods^43^,^44^,^45^,^46^,^47^. The regions co-occupied by six or more DBPs of the Core class showed more than twofold higher P300 signals than any of the other enhancers defined by the prior methods^43^,^44^,^45^,^46^,^47^ (Supplementary Fig. 5a), indicating that our enhancer-defining method outperforms the other widely used methods. Using the co-occupancy criterion of at least six DBPs, we defined 8,726 putative enhancers (Fig. 5a and Supplementary Dataset 4, see also Methods section) spanning an average of 1.6 kb long, and preferentially localizing to distal regions (median 25.9 kb from TSS). The majority of the enhancers we defined are within DNase I-hypersensitive regions (Fig. 5a), while a large portion of the enhancers defined by the other methods reside within non-accessible regions, which is indicative of false positives or inactive enhancers (Supplementary Fig. 5b). Similar to recent observations^26^, we found some enhancers spanning up to longer than 5 kb (253 enhancers; Fig. 5b). We observed a positive correlation between the co-occupancy of the Core class and the activity of their associated genes (hereafter, enhancer target genes; Fig. 5c) or the binding signal of the co-activators or mediators (Fig. 5d).

Recent reports suggested that H3K27me3 can serve as a marker for ‘poised’ enhancers^41^,^43^. Interestingly, we found that the H3K27me3 signature is almost completely depleted in the enhancers defined by the Core class. Since they still harboured both H3K4me1 and H3K27ac markers (Figs 1d and 5a), these findings suggest that the DBPs in the Core class exclusively occupy active enhancers, but not the poised enhancers in ES cells.

Although the Core class factors generally occupy distal cis-regulatory elements (Fig. 1b), some of the enhancers we defined reside near proximal promoters (Fig. 5e), and we found 376 genes associated with multiple enhancers: proximal (within 1 kb of TSS) and distal (within 20 kb of TSS) enhancers (Supplementary Dataset 5). Interestingly, these genes are more active than other genes solely associated with proximal enhancers, distal enhancers, super-enhancers^26^ or regulatory elements mapped by chromatin interaction analysis with paired-end tagging^48^ (Fig. 5f). Notably, these multiple enhancer-associated genes include many ES cell-specific regulators, such as *Oct4*, *Nanog*, *Sox2* and *Lin28a* (Supplementary Fig. 6a), and the loss of these genes preferentially leads to early developmental failure (Supplementary Fig. 6b), indicating that multiple enhancer-associated genes play important roles in controlling the identity of ES cells.

### Enhancer binding regulators are critical for CGI− genes

**Figure 6:**
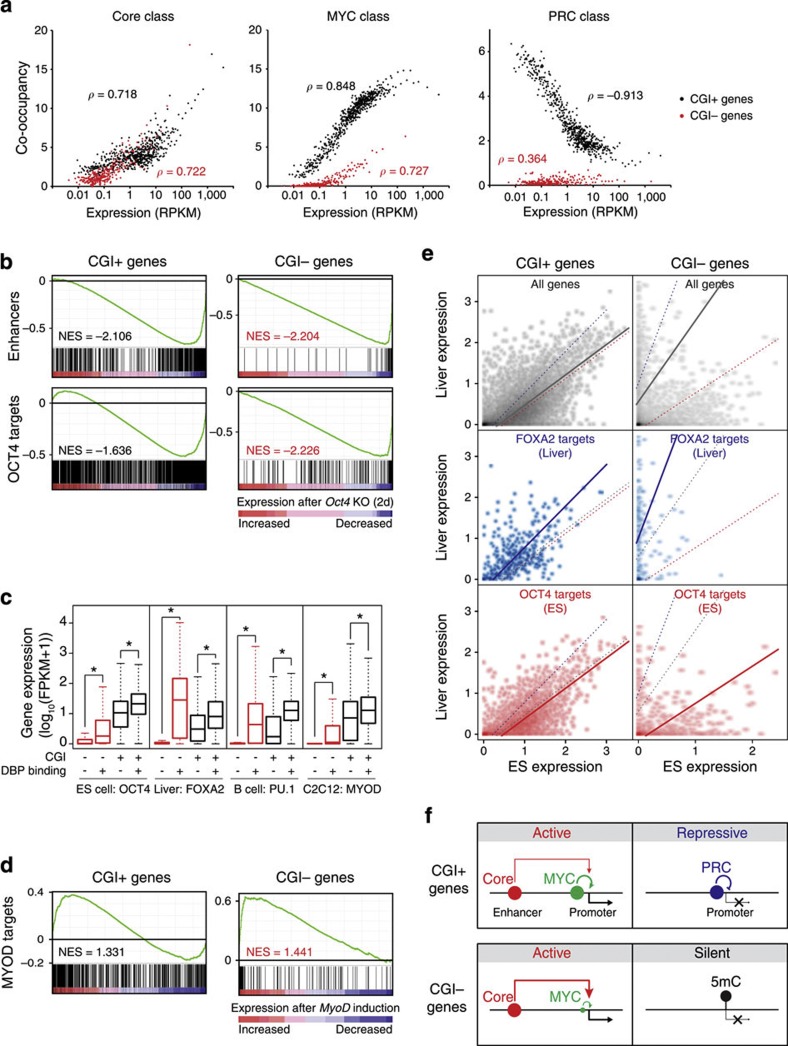
Enhancer-binding master regulators control the activity of tissue-specific CGI− genes in addition to general activity of CGI+ genes. (**a**) Correlations between the co-occupancy of DBPs in each class and the expression of CGI+ (black dots) or CGI− (red) target genes. Genes were sorted with expression levels and binned into every 20 genes. Each dot represents an average expression and co-occupancy of a group of genes. Gene groups with an average RPKM >0.01 are shown. *ρ* indicates Spearman correlation coefficient. (**b**) Gene set enrichment analyses (GSEA) using gene expression profile from conditional *Oct4* KO ES cells (GSE10477; ref. 62). Gene sets for enhancers (Fig. 5a) and OCT4 targets (±5 kb from TSS, GSM288354; ref. 13) were used. NES (normalized enrichment score). (**c**) Box plots showing the effect of tissue-specific enhancer-binding master regulators on their CGI+ (black boxes) or CGI− (red) targets. Specific cell types and tissue-specific master regulators are indicated. Target genes were assigned within±5 kb from the centre of the binding sites. FPKM, fragments per kilobase per million. *ρ<2.2e−16 (Wilcoxon signed-rank test). (**d**) GSEA using gene expression profile from *MyoD* induction in MEF cells (GSE6487)^64^ and MYOD targets (GSE36024)^59^. (**e**) Enhancer target gene expression (CGI+ or CGI− genes) between ES cells and liver cells. Expression of all genes (grey dots), FOXA2 targets (blue) and OCT4 targets (red) are shown in two-dimensional dot plots. Coloured trend lines are from least square linear regressions of each test. Gene expressions are shown as log_10_(FPKM+1). (**f**) A model of CGI-mediated global gene regulatory modes in ES cells. The MYC and PRC classes reciprocally regulate CGI+ genes, whereas the Core class regulates both CGI+ and CGI− target genes. A majority of CGI− genes are silent with DNA methylation while only a small subset of them are activated by the Core and MYC classes.

We showed that the reciprocal regulation between the MYC and PRC classes is predominant in CGI+ genes and controls the general activity of almost all CGI+ genes (Fig. 3). Since enhancers have been suggested as a critical regulatory component driving cell type- or tissue-specific gene expression^46^,^49^, we sought to elucidate the general roles of enhancers in CGI-dependent global gene regulation. As shown in Fig. 6a, the co-occupancy of the MYC and PRC classes showed strong positive and negative correlations, respectively, with the activity of only CGI+ genes. However, the co-occupancy of the Core class DBPs showed a similar positive correlation with the activities of the CGI+ and CGI− genes, indicating that the enhancers defined by the Core class are responsible for the activity of the CGI+ and CGI− target genes. Since CGI+ genes are generally active in various cell types (Fig. 2c), these results signify that enhancers play additive roles in the activity of their CGI+ target genes, but more decisive roles in the activity of CGI− target genes.

In order to further delineate the functional significance of enhancers in the regulation of CGI− genes, we examined the activity of enhancer-associated genes and targets of OCT4, a representative enhancer binding master regulator from the Core class, upon knockout of *Oct4* (Fig. 6b and Supplementary Fig. 7). The activities of the CGI+ and CGI− genes associated with the enhancers (upper) and OCT4 (bottom) were reduced upon two different perturbations, suggesting that enhancers (or OCT4) regulate both CGI+ and CGI− target genes. However, the extent of the expression decrease was significantly larger in the CGI− targets, confirming the more specific roles of enhancers in the CGI− target genes. We further examined the effects of enhancer binding master regulators on their targets in other cell types^46^: FOXA2 in liver cells, PU.1 in B cells and MYOD in C2C12 cells (Fig. 6c). For both CGI+ and CGI− genes, the overall activity of the genes associated with the master regulator was greater than the activity of the genes not associated with the master regulator. However, we consistently observed significantly more activity from the CGI− genes regulated by master regulators, whereas the non-target CGI− genes showed almost no detectable activity (Fig. 6c). Since CGI+ genes are generally active throughout various cell types (Fig. 2c), our results not only indicate that enhancers synergistically increase the activity of CGI+ target genes but also illustrate that enhancer-mediated transcriptional regulation is more critical for the activity of CGI− target genes. Consistent with this, ectopic expression of *MyoD* in fibroblasts led to significant global induction of the CGI− target genes, whereas the CGI+ target genes did not show a significantly unified response (Fig. 6d).

We further examined the activity of CGI+ and CGI− genes in ES versus liver cells. For CGI+ genes, the expression profiles of the ES and liver cells showed a strong positive correlation, indicating that the activity of many CGI+ genes is similar in both cell types. Although mildly skewed towards their tissue of origin, CGI+ genes that are targets of tissue-specific enhancer binding proteins (OCT4 for ES and FOXA2 for liver cells) also showed a similar expression pattern (Fig. 6e, left panel). For the CGI− genes, in contrast, the overall distribution of the gene expression profile was highly biased towards each axis, demonstrating the tissue-specific nature of the gene activity. Moreover, the CGI− targets of OCT4 or FOXA2 showed strong tissue-biased gene expression patterns (Fig. 6e, right panel). Taken together, our analysis strongly indicates that the enhancer binding proteins play roles in determining tissue-specific gene expression programs for the CGI− target genes, while these proteins play additive roles for the CGI+ target genes.

## Discussion

We performed a compendium analysis, integrating various DBP occupancies and chromatin status data sets, to elucidate global regulatory mechanisms in the context of CGI. After mapping multiple DBP classes based on co-occupancy, we revealed predominant reciprocal regulation between the MYC and PRC classes on CGI+ genes (Figs 2 and 3). We also showed the roles of the ES cell-specific enhancers defined by the Core class on CGI+ and CGI− genes, revealing the CGI-dependent modes of global gene regulation in ES cells and other cell types (Fig. 6f).

Unlike previous reports^19^,^30^, we found that a large portion of the CGI+ promoters remained bivalent even in differentiated tissues (Supplementary Fig. 3b), suggesting that the reciprocal regulation of CGI+ genes by the MYC and PRC classes may be a common feature shared in other cellular contexts. Although many inactive CGI+ promoters harbour H3K27me3 markers, the majority of the CGI− genes are silenced without a H3K27me3 marker in the ES cells and other differentiated tissues that we tested (Supplementary Fig. 4a,b). This ‘by-default silent’ state of the CGI− genes may allow the efficient management of limited resources in the cells, since most of these genes are not abundantly expressed or critical during the early developmental stages (Fig. 4c,d). This observation also indicates the necessity of the PRC-independent repression mechanism for the CGI− promoters. The unique mode of gene silencing on CGI− promoters possibly mediated by methyl-DNA-binding proteins will be of great interest for future studies (Supplementary Fig. 4c).

Consistent with previous reports showing the enrichment of tissue-specific DBP recognition motifs in the distal regulatory elements of CGI− genes^38^, we showed that the enhancer binding proteins govern tissue-specific CGI− gene expression programs. This is also consistent with the recent report of ‘super-enhancers’ occupied by master regulators that control cell type-specific gene expression^26^. The super-enhancers reported in ES cells, in turn, fall into the subset of enhancers we defined in this study (Supplementary Fig. 5c). Since tissue-specific CGI− gene expression programs turn on in parallel with terminal differentiation (Fig. 4e), further understanding of tissue-specific gene regulation mediated by enhancer binding master regulators, particularly in CGI− genes, would be helpful for developing more direct methods of controlling cell fates through induced trans-differentiation or direct reprogramming.

Taken together, our compendium analyses provide a conceptually unique perspective in understanding the global gene regulatory mechanisms. We used DBPs to define regulatory classes based on their target co-occupancy. Such defined classes then serve as powerful analytical tools in interrogating the global gene regulatory modes in ES cells with additional large data sets. Our analyses reiterate a unifying view of global transcriptional and epigenetic regulatory modes, especially incorporating CGIs as a crucial regulatory portal in determining general or tissue type-specific gene expression programs.

## Methods

### Cell cultures

Mouse J1 ES cell lines were maintained as described previously^24^. In detail, cells were maintained in ES medium (Dulbecco’s modified Eagle’s medium) supplemented with 15% fetal calf serum, 0.1 mM β-mercaptoethanol, 2 mM L-glutamine, 0.1 mM non-essential amino acid, 1% of nucleoside mix (100 × stock, Millipore), 1,000 U ml^−1^ recombinant leukaemia inhibitory factor (Chemicon) and 50 U ml^−1^ penicillin/streptomycin).

### ChIP-seq

ChIP assays were performed as described previously^24^. Flag-bio tagged ES cells (Supplementary Datasets 1,2) were fixed in 1% formaldehyde for 7 min at room temperature. The formaldehyde was quenched using final 125 mM glycine before harvesting cells. Sonicated chromatin extracts containing DNA fragments were immunoprecipitated using streptavidin-conjugated magnetic beads (Dynabeads MyOne Streptavidin T1). After washing and reverse crosslinking, purified ChIP DNA was applied for generation of sequencing libraries.

### Published ChIP-seq data analysis

ChIP-seq data from mouse ES cells published before 19 Dec 2012 were downloaded from Sequence Read Archive (SRA) in National Center for Biotechnology Information (NCBI) database. Downloaded data are listed in Supplementary Dataset 1 (DNA-binding proteins) and Supplementary Dataset 3 (chromatin status). FASTQ files were extracted with the SRA Toolkit version 2.1.6 and aligned using Bowtie 2.1.0 (ref. 50) onto the mouse genome (mm9, NCBI Build 37). For the identification of DBP-binding sites (Supplementary Dataset 1), model-based analysis for ChIP-seq peak caller (MACS 1.4.2; ref. 51) was used with a dynamic local lambda calculation and building shifting model with a *P* value cutoff of 1e−5. Regions containing specific histone marks (Supplementary Dataset 3) were identified with random Poisson distribution without a local lambda calculation or building the peak shape shifting model. For each peak calling, author-provided control ChIP-seq data were used to remove the background noise. For the experiments performed without any control ChIP reactions, sequencing data from whole-cell extract (GSM307154) were used as a universal control.

For the multiple ChIP-seq experiments for a single DBP (Supplementary Datasets 1,6), histone modification or DNase I hypersensitivity (Supplementary Dataset 3), ChIP-seq experiments carried out at different laboratories were treated as biological replicates and those from the same laboratory were treated as technical replicates. For technical replicates, only intersection regions of peaks from all replicates were used. On the other hand, for biological replicates, consensus peak regions from at least two experiments performed in different laboratories were used for the subsequent peak based analyses or identifications of DBP co-occupied regions (Fig. 1c (*y* axis), Fig. 1d (*y* axis), Fig. 3a (MYC and PRC class co-occupancies), Figs 3c and 5a (Core class co-occupancy), Figs 5a–d (*x* axis), Fig. 6a (*y* axis) and Supplementary Figs 1b,c, 3a,b and 5a,b (*x* axis)).

### Filtering low-quality ChIP-seq data

To monitor the quality of DBP ChIP-seq data, a signal-to-noise ratio (SNR) was calculated from duplicate read filtered bedGraph files generated by MACS for each ChIP-seq data as follows:

SNR=[area under signal curve within peak regions]/[area under signal curve outside of peak regions].

After filtering out all low-quality data with a stringent filtering criterion of >0.015 SNR, a total of 105 high-quality DBP ChIP-seq data out of 166 tested were used for the further analyses (Supplementary Dataset 2).

### CpG methylation analysis

For the mouse ES cells CpG methylation data used in Fig. 1c,d, bisulfite sequencing data from GSM1127953 (ref. 52) was used. In order to evaluate methylation status, FASTQ files were aligned with Bismark 0.10.0 and methylation was monitored with methylation extractor software^53^. Overall CpG methylation status in DBP peaks (Fig. 1c) or DBP co-occupied regions (Fig. 1d) were calculated by averaging methylation portions of all called CpG sequence within given regions.

### Comparison of DBP co-occupancy

The degree of co-occupancy between two DBPs shown in Fig. 1a was measured with the deviation of observed co-occupancy from the expected values determined by randomization. In detail, the binding sites of a DBP were randomly shuffled for 1,000 times with shuffleBed software in BEDtools suite v2.17.0 (ref. 54). In order to avoid bias arising from unmappable repetitive sequences, shuffling was performed only within the genomic regions occupied by at least one DBP. Moreover, to minimize the noise derived from the sex determining chromosome (chrY) used in ChIP-seq experiments, only the peaks in the X and somatic chromosomes were used. For each randomization, the length distribution of each DBP co-occupied region was monitored, and the *Z*-score representing the extent of co-occupancy was calculated from the mean and s.d. of expected length of co-occupied regions.

### Annotation of multiple DBP target loci and associated chromatin modification marks

For the identification of DBP co-occupied regions, DBP-bound regions within a class defined in Fig. 1a were merged using mergeBed software in BEDtools suite v2.17.0 (ref. 54). In order to examine the association of these DBP co-occupied regions with chromatin modification (Fig. 1d), histone marks were examined within the 300-bp regions from the centre of the merged peaks.

### Classification of promoters with CGI

CGI promoters were defined as regions containing any pre-defined CGI elements within a ±500-bp region from the TSSs of all annotated genes. In order to minimize false annotations, two CGI lists, determined by independent methods^18^,^55^ were used to map consensus CGI+ or CGI− promoter. In case of the gene containing multiple TSSs, only a single TSS showing the highest expression value in RNA-seq (GSM1005490; ref. 56) was used for the further analysis, to minimize the noise from rarely expressed minor transcripts.

### RNA-seq analysis

RNA-seq data from mouse ES cells (GSM1005490; ref. 56) were downloaded from SRA. FASTQ files were aligned to the mouse genome (mm9, NCBI Build 37) using TopHat^57^. For the ENCODE RNA-seq data sets^58^,^59^ (ES cells, adult liver, B cell and C2C12 in Fig. 6c), aligned bam files were downloaded and used. Gene expression was calculated as reads per kilobase per million (RPKM; single-end sequencing data, GSM1005490) or fragments per kilobase per million (FPKM; paired-end sequencing data, ENCODE) values using Cufflinks^60^. As ranges of RPKM values span over three orders of magnitude and tend to give high random multiplicative error in high expression values, expression values were converted into log_10_ scale (log_10_(RPKM+1)) to collapse the original range for graphical summarization.

### Microarray analysis

As a unified gene expression profile of diverse tissues and cell lines shown in Figs 2c and 4, microarray data from GNF (Genomics Institute of the Novartis Research Foundation) Mouse Gene Atlas V3 (GSE10246; ref. 34) were used. For the precise monitoring of expression values, raw data files (.cel files) were background-corrected and normalized with Robust Multi-Array expression measure using sequence information (GCRMA)^61^ methods to minimize the background signal originate from probe sequence or high GC contents. For genes with multiple probesets, only probes with maximal signal were used for the further analyses. For the clustering analysis shown in Fig. 4a,b, the average expression value from biological replicates was calculated in a natural scale, and each expression value was converted into a *Z*-score and clustered by unsupervised hierarchical clustering method using Xcluster software (http://www.stanford.edu/group/sherlocklab/cluster.html). For the expression data upon knock out of the *Oct4* gene shown in Fig. 6b and Supplementary Fig. 7, the microarray data set from GSE10477 (ref. 62) was downloaded and normalized with the Robust Multi-array Average^63^ method. For the expression data upon induction of MYOD in mouse embryonic fibroblast cell in Fig. 6d, the microarray data set from GSE6487 (ref. 64) was used

### Signal density normalization of ChIP-seq profiling

The read density of each ChIP-seq data was normalized to show the data in the same scale. For each ChIP-seq data, total area under the signal curve from duplicate read filtered bedGraph files generated by MACS was considered as one billion (1 × 10^9^). As a result, normalized signal density was shown as signal per billion as follows:

Normalized signal density=area under signal curve within region × 10^9^/total area under signal curve

### Representative ChIP-seq density profile analysis

For the ChIP-seq density profile analyses (Fig. 3a (excluding MYC and PRC class co-occupancies), Figs 3b and 5a (excluding Core class co-occupancy), Fig. 5d (*y* axis), Fig. 5e and Supplementary Figs 4a,c and 5a (*y* axis)), the following ChIP-seq data listed below were used as the representative one: H3 (GSM594580), H3K4me3 (GSM590111), H3K27me3 (GSM747539), H3K27ac (GSM851278), H3K4me1 (GSM845243), Pol II (GSM632040), OCT4 (GSM307137), NANOG (GSM307140), SOX2 (GSM288347), P300 (GSM723018), LSD1 (GSM637282), CHD7 (GSM558674), MED1 (GSM560348) and MED12 (GSM560345).

### Calculation of DBP-binding enrichment

In order to calculate the DBP-binding enrichment of a gene, log_10_ ratio of normalized tag density from a DBP ChIP-seq over the control ChIP-seq was used as follows:

DBP-binding enrichment=log_10_ {(normalized DBP ChIP signal in area+1)/(normalized control ChIP signal in area+1)}.

For the MYC and PRC class enrichment shown in Fig. 3b, median enrichment values among 18 MYC class DBPs and 5 PRC class DBPs (excluding TET1 and KDM2A from the PRC class in Fig. 1a) were used, respectively.

### TSS state mapping

Based on the existence^19^ (Supplementary Fig. 3b) of H3K4me3 and H3K27me3 marks within a 1 kb (±500 bp) region surrounding the TSSs, the promoters of all protein-coding genes were classified into four classes of histone status as follows; active (H3K4me3^+^, H3K27me3^−^), bivalent (H3K4me3^+^, H3K27me3^+^), repressive (H3K4me3^−^, H3K27me3^+^) and non-marked (H3K4me3^−^ and H3K27me3^−^).

### Gene set overlap test

The list of expression verified genes during development (Fig. 4c) was downloaded from Gene Expression Database on the MGI website (http://www.informatics.jax.org/expression.shtml)^39^. Functional annotation of genes (GOBP; Supplementary Fig. 6a) was downloaded from the Gene Ontology website (http://www.geneontology.org/)^65^. Genes involved in embryonic lethality (Fig. 4d) upon mutation and gene–phenotype relations (Supplementary Fig. 6b) were extracted from genotypes and mammalian phenotype annotations on the MGI website^40^. Tissue-specific gene lists (Fig. 4e) were downloaded from the Unigene website (http://www.ncbi.nlm.nih.gov/unigene). Gene set overlap tests were performed with hypergeometric distribution analysis using software R (http://www.r-project.org/). For multiple testing correction, hypergeometric probabilities were corrected by Benjamini and Hochberg^66^ false discovery rate. When the overlaps between two gene sets were overrepresented or underrepresented compared with expectations, they were considered as enriched or depleted, respectively.

### Definition of previously defined enhancers

Active and poised (Supplementary Fig. 5a,b) enhancers were determined as previously described^43^. In detail, P300 peaks containing H3K4me1, but not H3K4me3 marks, were divided into two groups based on the presence of H3K27me3. Previously identified active and poised enhancers^46^, as well as Enh elements^45^, were downloaded from the Supplementary Data provided by the authors; their genomic coordinates were converted into the mouse genome (mm9, NCBI Build 37) using Liftover software (http://genome.ucsc.edu/util.html). ChromHMM^47^ enhancer elements were identified by running ChromHMM software with 10 histone modifications and chromatin accessibility defined in Fig. 1c (excluding me-CpG).

### Mapping of enhancer/DBP target genes

To map the target genes of DBPs or enhancers, genes that are occupied by DBPs or enhancer (Supplementary Dataset 4) within ±5 kb from their TSSs were considered as the target genes (Figs 5c and 6a–e). For the mapping of gene that are regulated by multiple enhancers (Fig. 5f), genes containing both proximal (±1 kb from TSSs) and distal (±20 kb from TSSs) enhancers were used. Genes directly interacting with enhancers or OCT4-bound regions (Fig. 5f and Supplementary Fig. 7) were mapped using RNA pol II chromatin interaction analysis with paired-end tagging data from GSM1084137 (ref. 48).

### Data sets for tissue-specific enhancer binding DBPs

Target loci of tissue-specific DBPs in Fig. 6c were identified using following data. OCT4 (GSM288354; ref. 13), FOXA2 (GSM717562 and GSM717563; ref. 67), PU.1 (GSM537989; ref. 68) and MYOD (SRP001761; ref. 69).

## Additional information

**How to cite this article:** Beck, S. *et al.* CpG island-mediated global gene regulatory modes in mouse embryonic stem cells. *Nat. Commun.* 5:5490 doi: 10.1038/ncomms6490 (2014).

## Supplementary Material

Supplementary Information Supplementary Figures 1-7 and Supplementary References

Supplementary Dataset 1 166 genome-wide DBP occupancy data sets initially examined.

Supplementary Dataset 2 105 high-quality DBP occupancy data sets used for the DBP

Supplementary Dataset 3 Histone modification, DNase-seq, DNA methylation, and MBD

Supplementary Dataset 4 Enhancers co-occupied by multiple (6 or more) Core class DBPs in ES

Supplementary Dataset 5 376 genes regulated by multiple enhancers (both in proximal (1 Kb)

Supplementary Dataset 6 Multiple ChIP-seq experiments for a single DBP.
