# Supplementary material for: CpG island-mediated global gene regulatory modes in mouse embryonic stem cells
Source: Nat Commun. 2014 Nov 18;5:5490. doi: 10.1038/ncomms6490 (PMC4236720; doi:10.1038/ncomms6490)
Supplement: Supplementary Information — Supplementary Figures 1-7 and Supplementary References [file ncomms6490-s1.pdf]

SUPPLEMENTARY FIGURES

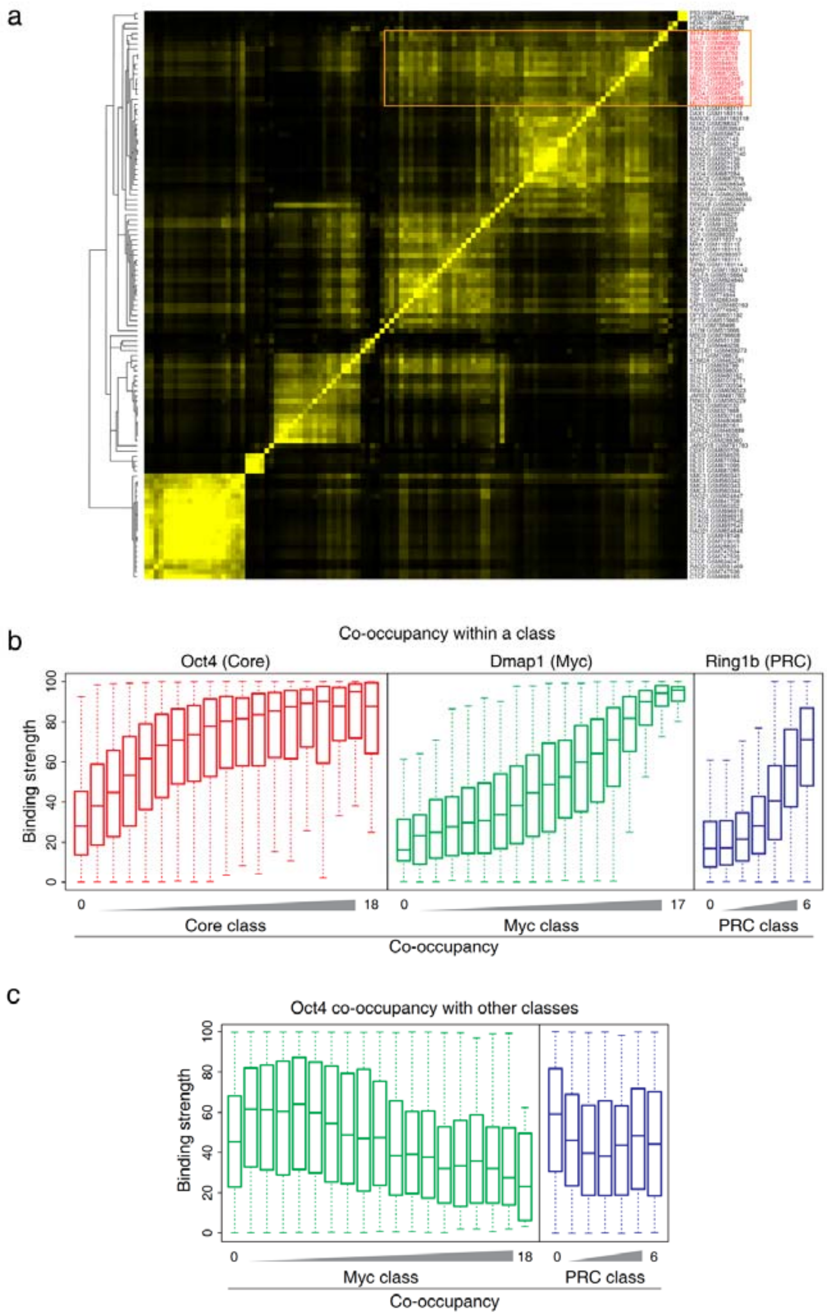

Supplementary Figure 1. Co-occupancy guided classification of DBPs.
(**a**) A correlation map from an unsupervised hierarchical clustering based on co-occupancy of DBPs including co-activators (P300, LSD1, and CHD7), and mediators (MED1 and MED12). This map is an extended version of **Figure 1a**. Putative enhancer-promoter linker DBPs are highlighted with an orange box.

(**b**) Box plots showing the correlation between the percentile binding strength of OCT4 (red), DMAP1 (green), or RING1B (blue) and target co-occupancy within the class indicated.
(**c**) Box plots showing the correlation between the percentile binding strength of OCT4 and its co-occupancy with DBPs in MYC (green) or PRC (blue) class.

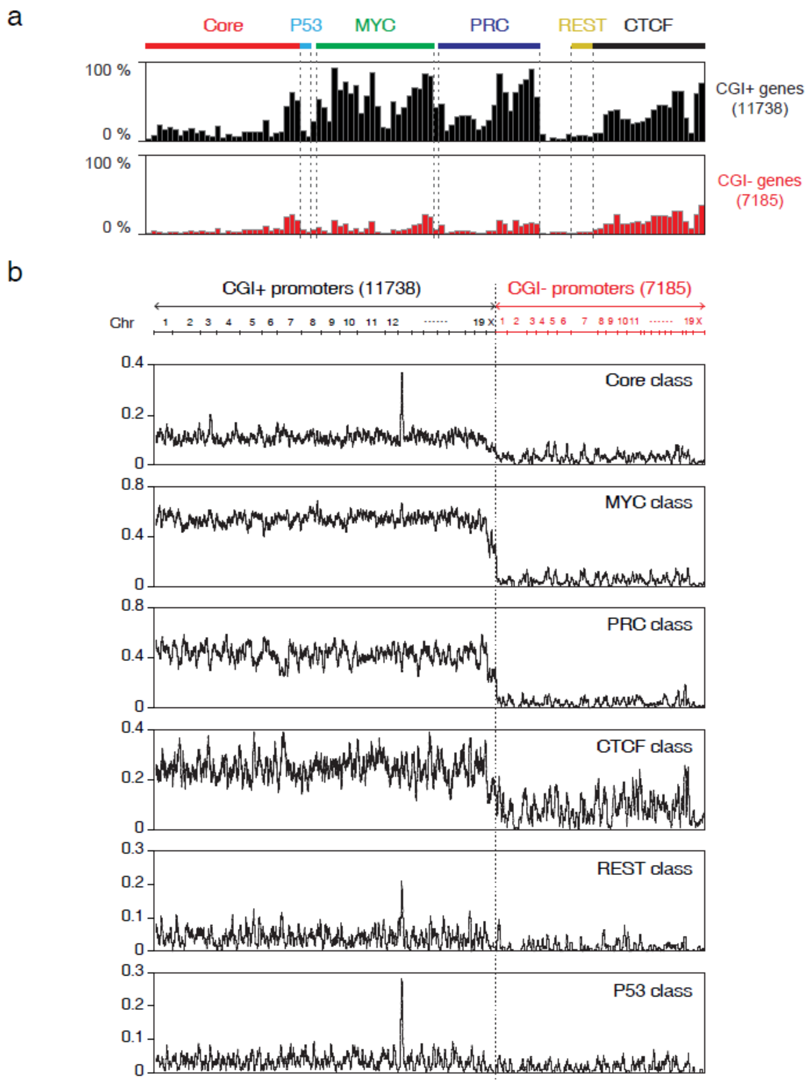

Supplementary Figure 2. CGI-dependent binding patterns of 6 distinct DBP classes.
(**a**) Portion of CGI+ and CGI- genes occupied by DBPs (± 5 Kb from TSS). DBPs are listed in the same order as in **Figure 1a**.
(**b**) The average binding of DBPs in each class within ±5 Kb region from the TSSs of CGI+ and CGI - genes is plotted using a moving average window (window size: 64, bin size: 1). Genes are in chromosomal order as **Figure 2a.**

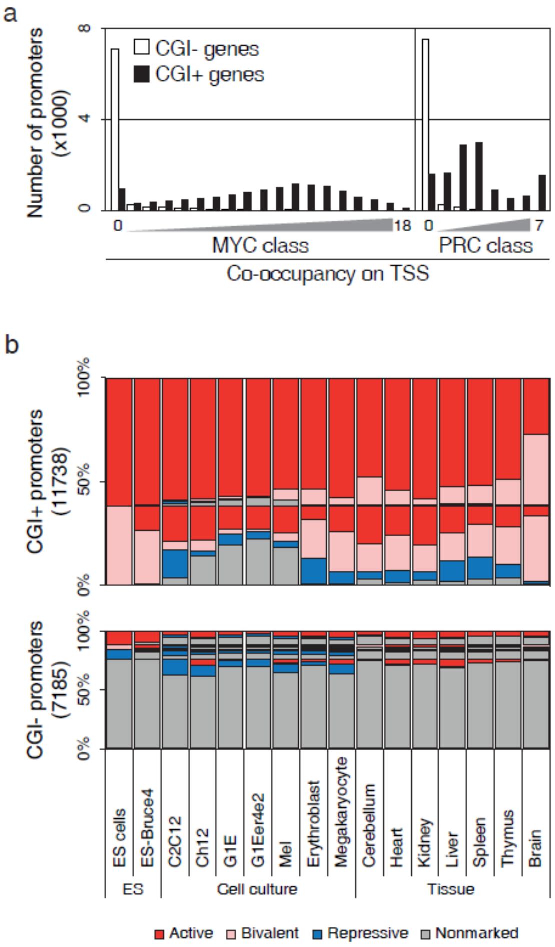

Supplementary Figure 3. Regulation of the MYC and PRC classes on CGI+ promoters.
(**a**) A bar chart showing the number of promoters (y-axis) co-occupied by indicated number (x-axis) of DBPs in the MYC or PRC class.
(**b**) Distribution of the 4 chromatin statuses including active (H3K4me3+/H3K27me3-), bivalent (H3K4me3+/H3K27me3+), and repressive (H3K4me3-/H3K27me3+) histone marks, as well as nonmarked (H3K4me3-/H3K27me3-) on CGI+ and CGI- promoters (± 500 bp regions from TSSs) in various tissues and cell types.

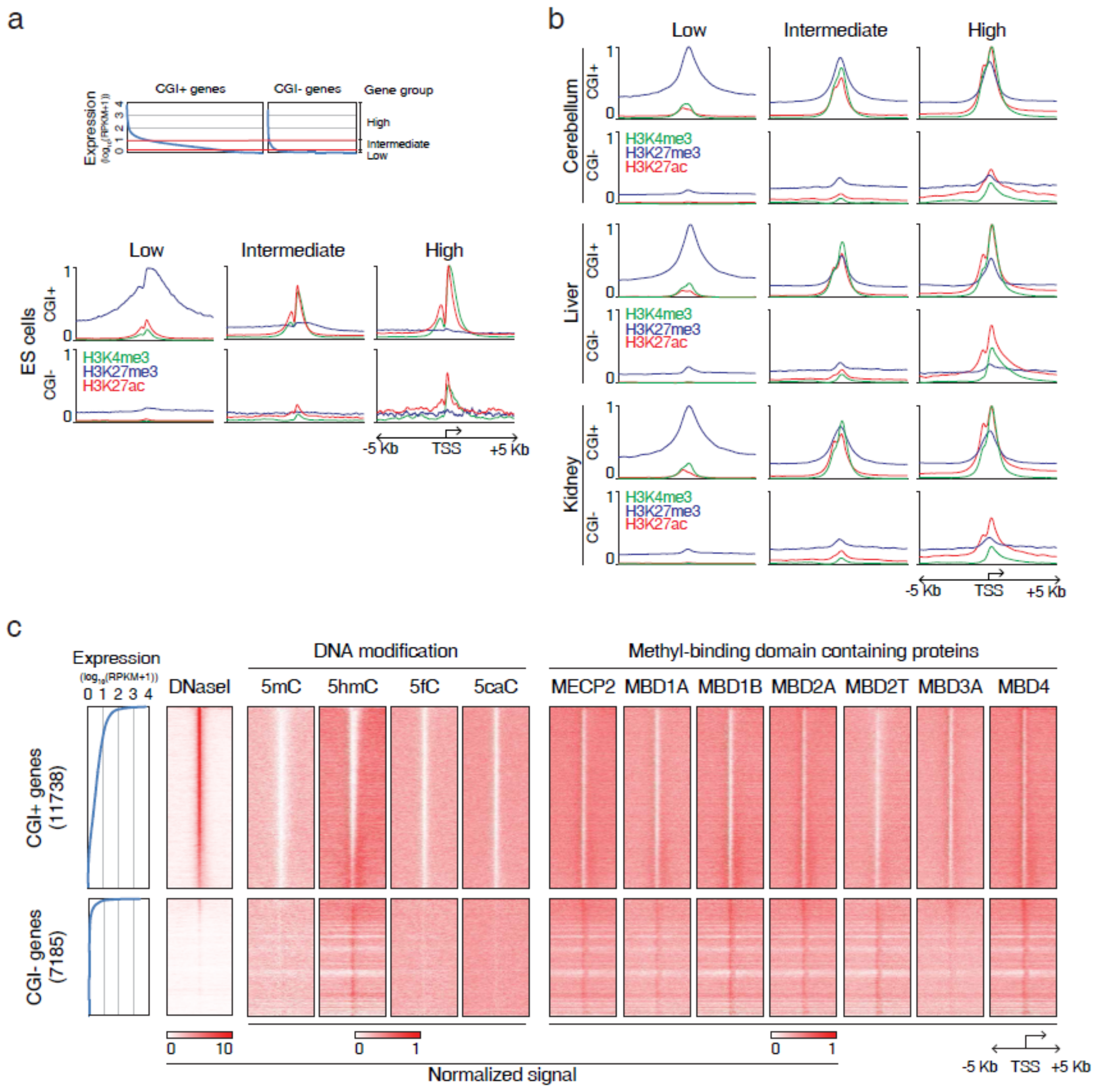

Supplementary Figure 4. DBPs and histone marks involved in the repression of CGI+ promoters.

(**a**) CGI+ and CGI- genes were classified into three groups based on their expression values (low, intermediate, and high) in ES cells. Histone modifications associated with the indicated groups are shown (± 5 Kb regions from the TSSs).

(**b**) CGI+ and CGI- genes were classified into three groups based on their expression values (low, intermediate, and high as in **Supplementary Fig. 4a**) in three differentiated tissues (cerebellum, liver, and kidney). Histone modifications associated with the indicated groups are shown (± 5 Kb regions from the TSSs).

(**c**) Heatmaps showing DNA modification signals and occupancy of methyl binding domain (MBD) containing proteins within ± 5 Kb from the TSSs in ES cells[1,2]. All protein coding genes were ranked by their expression levels in ES cells (left panel) and corresponding modifications or factor binding signals are shown.

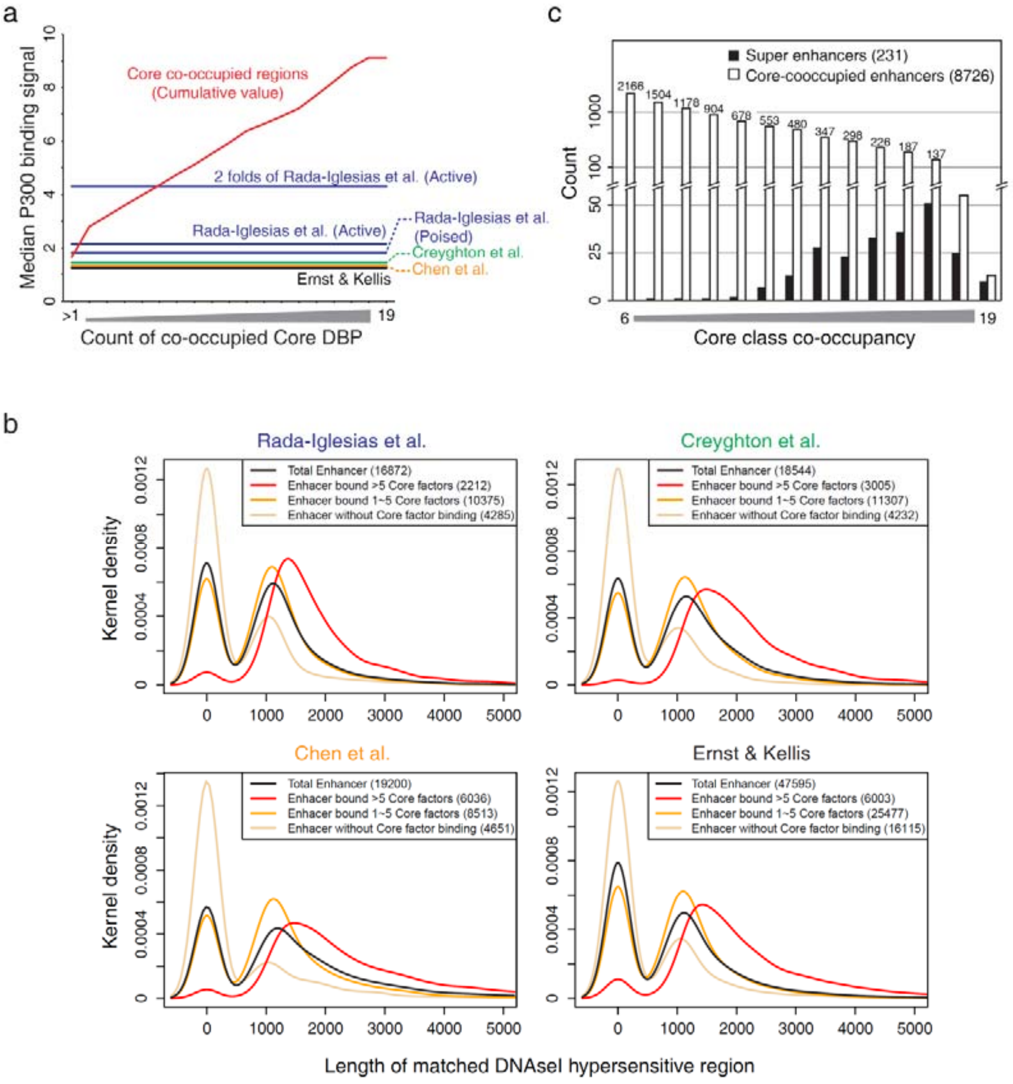

Supplementary Figure 5. Comparison of enhancers defined by Core class co-occupancy and other annotation methods.

(**a**) Median signals of P300 occupancy within enhancer loci defined by the co-occupancy of the Core class. Previously published methods were also tested for comparison[3-7].

(**b**) Kernel density distribution of matched DHR length in previously annotated enhancer elements. Note that annotated enhancers without DBP bindings are largely depleted with DHR, implying their non-functionality.

(**c**) Numbers of the putative enhancers defined by the Core class (6 or more DBPs). Previously defined super enhancers[8] are shown for comparison; all 231 super enhancers are part of the enhancers defined by the Core class co-occupancy.

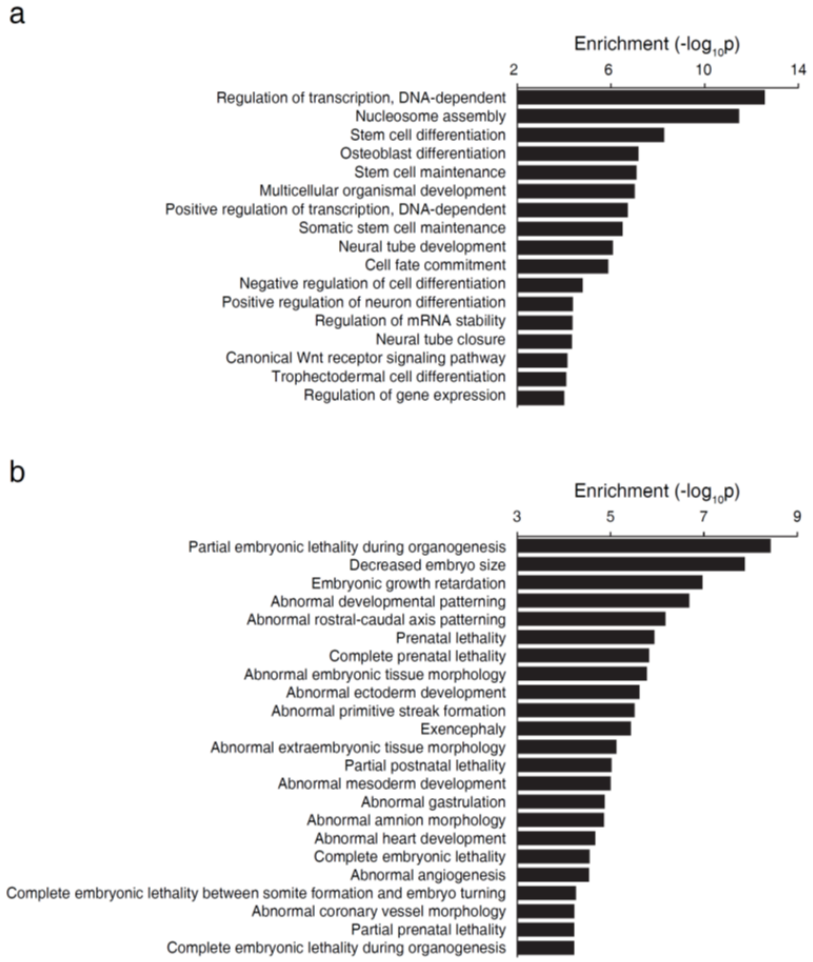

Supplementary Figure 6. Functions of genes associated with multiple enhancers.
(**a,b**) Enrichment of GOBP (Gene Ontology Biological Process) terms (**a**) and phenotypic defects related terms (**b**). All genes regulated by multiple enhancers in ES cells were tested. p-values were calculated from gene set overlap test using a hypergeometric distribution.

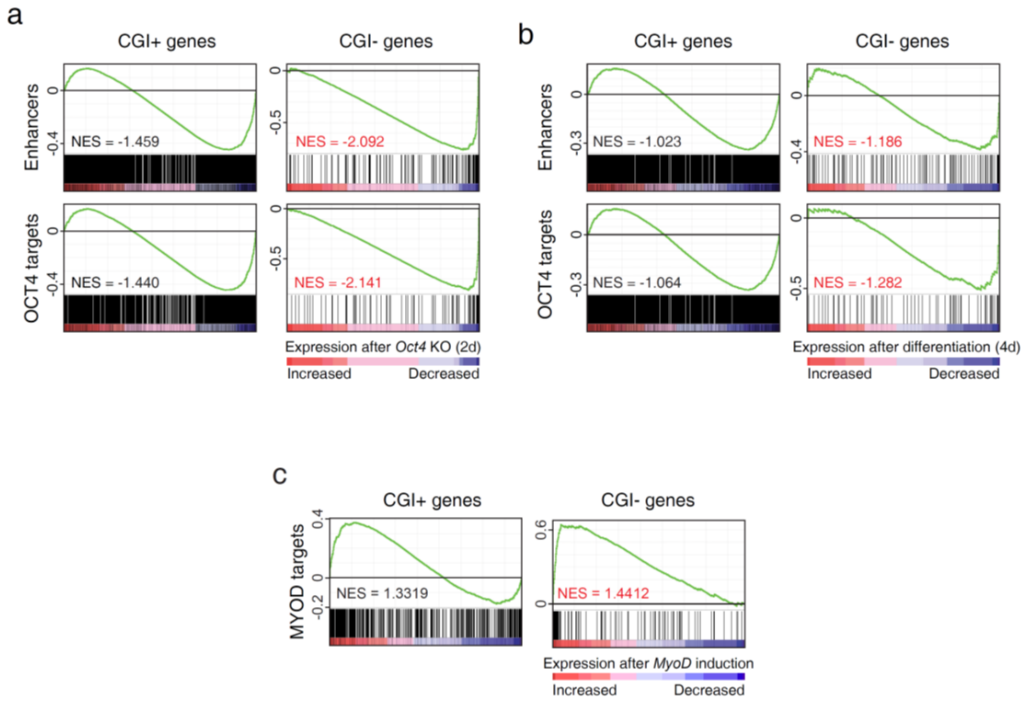

Supplementary Figure 7. Genes physically interacting with enhancers or OCT4.
Gene set enrichment analyses (GSEA)[9] using gene expression profile data from conditional *Oct4* KO ES cells (GSE10477)[10]. Target genes indicate the genes physically interacting with enhancers or OCT4 binding loci determined by ChIA-PET[11]. NES stands for Normalized Enrichment Score of GSEA.

SUPPLEMENTARY REFERENCES

1. Baubec, T., Ivánek, R., Lienert, F. & Schübeler, D. Methylation-dependent and -independent genomic targeting principles of the MBD protein family. *Cell* **153**, 480-492 (2013).
2. Shen, L. *et al.* Genome-wide analysis reveals TET- and TDG-dependent 5-methylcytosine oxidation dynamics. *Cell* **153**, 692-706 (2013).
3. Chen, C.Y., Morris, Q. & Mitchell, J.A. Enhancer identification in mouse embryonic stem cells using integrative modeling of chromatin and genomic features. *BMC Genomics* **13**, 152 (2012).
4. Creyghton, M.P. *et al.* Histone H3K27ac separates active from poised enhancers and predicts developmental state. *Proc Natl Acad Sci U S A* **107**, 21931-21936 (2010).
5. Ernst, J. & Kellis, M. ChromHMM: automating chromatin-state discovery and characterization. *Nat Meth* **9**, 215-216 (2012).
6. Rada-Iglesias, A. *et al.* Epigenomic annotation of enhancers predicts transcriptional regulators of human neural crest. *Cell stem cell* **11**, 633-648 (2012).
7. Rada-Iglesias, A. *et al.* A unique chromatin signature uncovers early developmental enhancers in humans. *Nature* **470**, 279-283 (2011).
8. Whyte, Warren A. *et al.* Master transcription factors and mediator establish super-enhancers at key cell identity genes. *Cell* **153**, 307-319 (2013).
9. Subramanian, A. *et al.* Gene set enrichment analysis: A knowledge-based approach for interpreting genome-wide expression profiles. *Proc Natl Acad Sci U S A* **102**, 15545-15550 (2005).
10. Endoh, M. *et al.* Polycomb group proteins Ring1A/B are functionally linked to the core transcriptional regulatory circuitry to maintain ES cell identity. *Development* **135**, 1513-1524 (2008).
11. Zhang, Y. *et al.* Chromatin connectivity maps reveal dynamic promoter-enhancer long-range associations. *Nature* **504**, 306-310 (2013).
